# The Role of Microbiota and Immunobiotics in Granulopoiesis of Immunocompromised Hosts

**DOI:** 10.3389/fimmu.2017.00507

**Published:** 2017-05-08

**Authors:** Susana Salva, Susana Alvarez

**Affiliations:** ^1^Immunobiotechnology Laboratory, CERELA-CONICET, Tucuman, Argentina; ^2^Applied Biochemistry Institute, Universidad Nacional de Tucumán, Tucuman, Argentina

**Keywords:** immunobiotics, granulopoiesis, immunocompromised hosts, respiratory infections, *Lactobacillus rhamnosus* CRL1505

## Abstract

The number of granulocytes is maintained by a regulated balance between granulopoiesis in the bone marrow and clearance and destruction in peripheral tissues. Granulopoiesis plays a fundamental role in the innate immune response. Therefore, factors affecting the normal granulopoiesis lead to alterations in innate defenses and reduce the resistance against infections. In this study, we give a description on recent advances regarding the molecular and cellular events that regulate steady-state and emergency granulopoiesis, which are crucial processes for the generation of protective innate immune responses. Particular attention will be given to emergency granulopoiesis alterations in immunosuppression states caused by malnutrition and chemotherapy. The role of microbiota in maintaining a steady-state granulopoiesis and the immunological mechanisms involved are also discussed. Moreover, we describe the findings of our laboratory demonstrating that the dietary supplementation with immunobiotics is an interesting alternative to improve steady-state and emergency granulopoiesis, the respiratory innate immune response, and the resistance against respiratory pathogens in immunocompromised hosts.

## Introduction

The microbiota is a complex community of bacteria, fungi, archaea, and viruses that colonize the mucosal surfaces and skin of the human body (1). The gut microbiota is typically integrated by bacteria and specifically by members of the divisions *Bacteroidetes* and *Firmicutes* (2). However, each individual’s microbiota is unique, and its composition depends on multiple factors, such as, diet, lifestyle, host genetic, use of antibiotics, and environment (3–5). The gut microbiota plays a fundamental role in the health maintenance of its host. In particular, we highlight that microbiota is able to control immunity in distant tissues through its capacity to regulate hematopoiesis at primary immune sites as the bone marrow (BM) (6). On the other hand, the immunomodulatory probiotic lactic acid bacteria (immunobiotics) are capable to improve the recovery of myeloid cells production affected by malnutrition or chemotherapy, and to increase the immune response against bacterial pathogens (7, 8). The mechanisms for systemic immunomodulation by the microbiota provide a probable mechanism for immunobiotics activity, demonstrating that translocated microbial products benefit the host by enhancing systemic innate immune function.

In this mini review, we examine the interaction between microbiota and immune system, and how this crosstalk affects the maintenance of a steady-state granulopoiesis that is crucial for the generation of protective innate immune responses. We also revise the alterations of emergency granulopoiesis in immunosuppression states caused by malnutrition and chemotherapy. In addition, we discuss the research of our laboratory demonstrating that dietary supplementation with immunobiotics is an interesting alternative to improve steady-state and emergency granulopoiesis, respiratory innate immune response, and resistance against respiratory pathogens in immunocompromised hosts.

## Granulopoiesis and Its Regulation by the Gut Microbiota

Granulocytes are key players of the innate immune response. They are short-lived cells, and their number is kept by a balance between BM granulopoiesis and peripheral tissues’ clearance and destruction (9). These cells are continuously generated in steady-state conditions from long-lived self-renewing hematopoietic stem cells (HSCs) that give rise to short-lived HSCs and multipotent progenitors (MPPs) in BM. MPPs differentiate into common lymphoid and myeloid progenitors (CMP). CMPs give rise to granulocyte-macrophage (GMP), megakaryocyte-erythrocytes, and dendritic cell progenitors. Neutrophils, monocytes, as well as other granulocyte populations derive from GMPs (10). In front of an infectious challenge, neutrophils are recruited in large numbers to the infected tissues, the hematopoietic system must rapidly respond to the demand of these cells by turning from the steady-state to an emergency granulopoiesis (11).

The tissue macrophages activate the LRX family transcription factors during the ingestion of apoptotic neutrophils in steady-state granulopoiesis. This, in turn, suppresses the pro-inflammatory cytokines’ production (12). Those macrophages decrease their production of interleukin (IL)-23 and thereby reduce the stimulus for IL-17 production by innate lymphoid cells, natural killer T cells, γδ-T cells, or Th17 cells. The reduced IL-17 levels account for low G-CSF expression (13). Involvement of IL-23/IL-17/G-CSF axis in regulation of granulopoiesis was confirmed in several independent murine models (14) and human studies (15). Moreover, steady-state neutrophil homeostasis is G-CSF-dependent and regulated through pattern-recognition receptors (PRRs), thereby directly linking Toll-like receptor (TLR)-triggering to granulopoiesis (14). Microbiota-derived components, such as LPS *via* TLR4/MyD88 signaling, induced intestinal IL-17 production and increased plasma levels of G-CSF leading to granulocytosis (16). Studies using antibiotics-treated and germ-free (GF) mice showed a decrease in GMPs in BM, and a lower number of neutrophils in the periphery (16–20). Furthermore, it was reported that a live complex flora is needed to restore granulopoiesis completely (21, 22). By using MyD88-deficient mice, it was shown that MyD88-dependent TLR signaling induced by microbiota can impact on the early hematopoietic development and terminal myeloid differentiation (20, 21) (Table 1).

**Table 1:** **Regulation of granulopoiesis by microbiota and immunobiotics**.

Experiemental models	Outcome
**Microbiota-granulopoiesis crosstalk**	

Polymyxin treated and germ-free (GF) mice (18)	Decreased levels of splenic and bone marrow (BM) progenitor cells forming GM-CFU colonies

Kanamycin-treated mice (17)	Decreased numbers of granulocytes in the BM and blood

GF mice Antibiotic-treated mice Nod1-deficient mice (23)	Translocation of peptidoglycan from the gut to neutrophils in the BM Peptidoglycan concentrations in sera correlate with neutrophil function Antimicrobial capacity of neutrophils depends on recognition of microbiota-derived peptidoglycan from Gram-negative bacteria *via* the pattern-recognition receptor Nod1 but not Nod2 orTLR4 Consistent with impaired innate priming Nod1-deficient mice were unable to control *Streptococcus pneumoniae* early sepsis

GF mice *TLR4^−/−^* mice *TRIF^−/−^* mice *MyD88^−/−^* mice (14)	Total lack of microbial colonization is associated with decreased neutrophil numbers and decreased G-CSF levels in the steady state *In vivo* feedback is impaired in *TLR4^−/−^* and *TRIF^−/−^*, but not *MyD88*^−/−^ animals Steady-state neutrophil homeostasis is G-CSF-dependent and regulated through pattern-recognition receptors, thereby directly linking toll-like receptor (TLR)-triggering to granulopoiesis

MyD88-deficient mice (20)	Loss of MyD88 reduce numbers and proliferation rate of hematopoietic stem cells in the BM

GF mice (27)	Reduction of neutrophil recruitment to the peritoneal cavity in response to diverse stimuli including microbial components and sterile ligands Microbiota-derived signals *via* a MyD88-dependent pathway are required to precondition the neutrophil inflammatory response

Antibiotic-treated neonatal mice GF neonatal mice (16)	Decreased numbers of circulating and BM neutrophils and lower granulocyte-macrophages in the BM Decreased number of interleukin (IL)-17-producing cells in the intestine and production of G-CSF Increased susceptibility to *Escherichia coli* K1 and *Klebsiella pneumoniae* sepsis, which could be partially reversed by administration of G-CSF. Microbiota-derived components, such as LPS *via* TLR4/MyD88 signaling, induced IL-17 production mainly by group 3 innate lymphoid cells in the intestine and increased plasma levels of G-CSF leading to granulocytosis

GF mice (19)	Damage of differentiation of specific myeloid cell progenitors of both yolk sac and BM origin Innate immune defects lead to impaired early responses to pathogens

GF mice (21)	Complexity of the intestinal microbiota correlates with the number of BM myeloid cells Transfer of sterile filtered, boiled serum from colonized mice was sufficient to expand the BM myeloid cell pool of GF mice in a MyD88/Toll-IL-1 receptor-containing adaptor molecule-dependent manner, indicating that heat-stable, circulating, commensal-derived products may be responsible for maintaining steady-state myelopoiesis levels

Antibiotic-treated mice (28)	Impairment of resistance to lung infection with *K. pneumoniae* and reduction of ROS-mediated bacterial killing by alveolar macrophages and required Nod1- and Nod2- but not TLR-dependent signaling

GF mice Antibiotic-treated mice (29)	Reduction steady-state numbers of tissue-resident and BM-derived phagocytes rendered GF and antibiotic-treated mice susceptible to acute systemic *Lysteria monocytogenes* infection

Neonatal non-obese diabetic mice (22)	The gut microbiota enriched in *Staphylococcus* in neonatal non-obese diabetic mice, correlated with increased immature granulocytes in the liver and spleen

**Immunobiotics-granulopoiesis crosstalk**	

Protein-malnourished mice (33)	Protein malnutrition altered B cell development in BM. The treatment of malnourished mice with *Lactobacillus rhamnosus* CRL1505 was able to induce a recovery of B cells that would explain its ability to increase immunity against infections

Protein-malnourished mice (35)	Repletion of malnourished mice with supplemental *Lactobacillus rhamnosus* improved neutrophils recruitment, phagocytic activity, and resistance against pneumococcal infection

Protein-malnourished mice (7)	Protein-malnutrition impaired the emergency myelopoiesis induced by the generation of the innate immune response against pneumococcal infection Repletion of malnourished mice with *Lactobacillus rhamnosus* CRL1505 was able to accelerate the recovery of granulopoiesis and improve innate immunity

Chemotherapy-treated mice (8)	*Lactobacillus casei* CRL431 and *Lactobacillus rhamnosus* CRL1506 accelerated the recovery of Cy-caused myelosuppression and the recovery of the immune response against the opportunistic pathogen *Candida albicans*

Chemotherapy-treated mice (53)	*Bifidobacterium longum* BB536 increased resistance to sepsis caused by an intestinal infection of *Pseudomonas aeruginosa* in mice treated with Cy

Chemotherapy-treated mice (42, 54)	*Lactobacillus plantarum* HY7712 as *Lactobacillus casei* HY7213 accelerated the recovery of Cy-induced immunosuppression by immunopotentiating NK cells and cytotoxic T lymphocytes derived from BM and spleen, in addition to restoring the phagocytic capacity of peritoneal macrophages

Chemotherapy-treated mice (55)	*Lactobacillus plantarum* stimulated the proliferation of splenocytes from mice immunocompromised by Cy treatment in response to LPS

During emergency granulopoiesis, pathogen-associated molecule patterns (PAMPs) are detected by PRRs of innate immune system. In addition, bacteria and bacteria-derived products (e.g., LPS) are sensed by TLR-expressing endothelial cells. Consequently, the granulopoiesis and the neutrophils’ release into the circulation are produced by the increased amounts of G-CSF and GM-CSF (11). A large body of evidence suggests that circulating microbiota-derived products or pathogens may reach the BM or extramedullar sites, where they can be directly sensed by HSPCs and committed myeloid progenitors (23, 24). On the other hand, circulating HSCs can encounter bacteria or their products in the periphery before re-entering the BM (10). LPS-sensing by hematopoietic cells is dispensable for the induction of emergency granulopoiesis. TLR4 and MyD88 expression of non-hematopoietic cell type is absolutely required for this process (25, 26). Thus, levels of growth factors determine the rate at which neutrophils are induced the proliferation and differentiation of neutrophil precursors by JAK–STAT pathways. In this context, transcription factor C/EBP-α regulates steady-state granulopoiesis, whereas C/EBP-β is critical for triggering emergency granulopoiesis in response to GM-CSF (11). On the other hand, recent studies demonstrate that microbiota priming is required for neutrophil extravasation to injured tissues after inflammatory stimuli (27). In addition, antimicrobial capacity of neutrophils was shown to be dependent on the recognition of microbiota-derived peptidoglycan from Gram-negative bacteria *via* Nod1 but not Nod2 or TLR4 (23). An example of the systemic effect of gut microbiota on granulopoiesis during infection has been provided by a research work demonstrating that early innate resistance to *Klebsiella pneumoniae* lung infection was impaired in microbiota-depleted mice, and that the peptidoglycan translocated from the gut was able to modulate the systemic innate immunity (28). Therefore, factors affecting the normal granulopoiesis lead to alterations in innate defenses and reduce the resistance against pathogens (23, 28, 29) (Table 1).

## Granulopoiesis and Malnourished Hosts

Granulopoietic homeostasis requires an important cellular renewal, because of the cells’ generation and death. Approximately 0.5–1.0 × 10^11^ granulocytes are generated each day during steady-state conditions in adults (11). In contrast to local infection that can be contained by the innate immune response, in severe infections, the emergency granulopoiesis is triggered and neutrophilia occurs. Therefore, the hematopoietic system is capable of rapid adaptation when augmenting cellular output several-fold levels to respond to the higher demand for neutrophils (10). Steady-state growth and development, physical activity, and response to serious illness are affected by nutritional status (30). In agreement to several research works, we have established that malnutrition affects the hematopoietic tissue that has a high turnover rate and cell proliferation, inducing a damage of blood cells production and causing hypoplasia and histological alterations of BM (31–33). This is characterized by a reduction of hematopoietic space, which is occupied by components of extracellular matrix (31, 33). These histological alterations may be responsible for the damage of the hematopoietic niches, which may influence the crosstalk between hematopoietic cells and the growth factors that regulate the granulopoiesis. In line with Borelli et al. (34), a reduction of GMPs was observed in BM of malnourished mice that could explain the reduction of myeloid cells of BM and blood (7, 35). Thus, nutritional deficiencies affect hematopoiesis, leading to an immunocompromised condition (35).

The relationship between malnutrition and infection can be viewed under two aspects: malnutrition compromising host defense, or infections either aggravating a previously existing deficient nutritional status or triggering malnutrition through disease pathogenesis. It was described that malnutrition alters both innate and adaptive immune responses as consequence of multiple abnormalities induced in the immune system (36). Herrera et al. (7) demonstrated that protein-malnutrition significantly reduces the capacity to recruit neutrophils into infected lungs and that this effect could be related to impairment in granulopoiesis. Several factors could be involved in the impairment of emergency granulopoiesis in malnourished mice, in addition to those mentioned above. CXCL12 expression in response to pneumococcal infection in BM requires special attention. The HSCs homing into BM is regulated by CXCL12 and their receptor CXCR4 (9). There is no change in the expression of CXCL12 during an infection in malnourished mice, which could be a mechanism for the preservation of HSCs in the BM. Malnutrition also impairs the expression of both GM-CSF and IL-1 in BM and contributes to the altered emergency granulopoietic response (7). It is known that the steady-state and emergency granulopoiesis are directed by GM-CSF while the BM stromal cells function is to support hematopoiesis mainly regulated by IL-1 (37). Hence, the neutrophilia induced by infection or inflammation is assisted by both GM-CSF and IL-1, which in turn accelerates granulopoiesis by expanding MPP and CMP compartments (38). Thus, the reduced capacity of malnourished hosts to develop adequate levels of GM-CSF may explain BM’s defective response against an infectious challenge.

On the other hand, there is a great increase in the number of patients with secondary immunodeficiencies related to chemotherapeutic treatments. Cyclophosphamide (Cy) is a drug widely used as an antineoplastic alkylating agent because of its significant therapeutic range and broad spectrum of activity to treat different types of cancer (39). For the World Health Organization, Cy is one of the essential medicines needed in a health system (40). However, this drug induces serious side effects, such as apoptosis and necrosis in BM cells (41), alterations of basal and emergency hematopoiesis, immunosuppression (8, 42), increased susceptibility to infections (8, 43), and even change of intestinal microbiota composition (44). Because of the increased susceptibility to infections, chemotherapy is commonly used in combination with antibiotics in cancer therapy (45). Paradoxically, the consequent propagation of antibiotic resistance among pathogens and depletion of intestinal microbiota lead to increased vulnerability of these patients. For these reasons, it is vital to support treatments aimed at recovering the hematopoietic capacity to increase the efficiency of the immune response triggered in infectious hematopoietic alternative resources processes.

## Can Oral Administration of Immunobiotics Regulate Granulopoiesis?

In the last years, a number of research project were meant to find alternative treatments to favor hematopoiesis, improve immunity, booster anticancer effects, and clear anticancer drugs (46). A long list of health benefits has been described for immunobiotics, are likely to modulate and enhance immunity functions in malnourished mice (8, 47–49) (Table 1).

Therefore, when the diet induced *Lactobacillus casei* CRL431 or *Lactobacillus rhamnosus* CRL1505, the recovery of the respiratory immunity in immunocompromised hosts was reduced from 21 to 7 days (32, 50, 51). Furthermore, the supplementation of repletion diet with immunobiotics induced recovery of mielopoyesis and normalization of emergency granulopoiesis in response to pneumococcal infection (7, 32). We demonstrated that the treatments with immunobiotics were efficient to recover the architecture of BM tissue, subendosteal epithelium, and BM cellularity altered by malnutrition (7). Moreover, the administration of *L. rhamnosus* CRL1505 induced the growth of mitotic pool cells, mature myeloid cells, and neutrophils in BM. Although the mechanisms involved are not completely elucidated, it is known that cell wall components reach the gut mucosa and from there to circulation during colonization of gut mucosa by commensal bacteria or probiotics. Indeed, the detection of peptidoglycan in the neutrophil fraction shows that it can accumulate in the BM (23). Considering these findings, a probable mechanism for the immunobiotic activity of the microbiota was observed, demonstrating that microbial products favor the systemic innate immune function of the host.

On the other hand, it was described that some immunobiotics can influence ILs levels in blood, which agree with our findings demonstrating the capacity of immunobiotics to normalize the levels of TNF-α, IL-1β, IL-4, IL-6, and IL-10 in malnourished mice (52). It is probably that the changes in ILs levels induced by immunobiotics could influence on the normalization of granulopoiesis. Moreover, we demonstrated for the first time that dietary supplementation with immunobiotics can modulate the production of GM-CSF in infected lungs and its expression in the BM. Moreover, immunobiotics modulate the CXCR4/CXCL12 signaling axis, which is associated with the recovery of hematopoiesis induced by *L. rhamnosus* CRL1505 (7). The detailed study of the mechanisms that explain the influence of immunobiotics on the regulation of granulopoiesis in BM is an interesting topic for future investigations.

Some works have described beneficial effects of immunobiotics on myelosuppression and immunosuppression in Cy-treated mice, although no deep mechanistic studies were performed (8, 42, 53–55) (Table 1). Taking into consideration the capacity of *L. casei* CRL431 and *L. rhamnosus* CRL1506 to modulate hematopoiesis in malnourished mice, we also aimed to evaluate the ability of these immunobiotic strains in Cy-treated mice. We showed that preventive treatment with immunobiotics is capable to increase GMPs in BM (CD34^+^ and CD34^+^Gr-1^+^ cells), which enables a prompt recovery of peripheral blood neutrophils after Cy-administration (8). These immunobiotic treatments were also able to improve recruitment of phagocytic cells to the site of infection and increase resistance against *Candida albicans* (8). Further studies to evaluate the mechanisms involved in these activities are needed. However, these results support the idea that immunobiotic strains can improve the recovery from Cy-immunosuppression, enhancing myeloid population in BM. Therefore, immunobiotics can serve as alternatives to reduce the immunosuppression in patients treated with chemotherapy drugs.

## Concluding Remarks

This review exposes wide evidence that the gut microbiota regulates granulocyte homeostasis, and therefore influences the host’s innate immune response. Additionally, research from the last years demonstrated that the oral administration of immunobiotics improves the recovery of steady-state granulopoiesis and stimulate the emergency granulopoiesis in malnourished and Cy-immunocompromised host. Future research is needed in order to elucidate the mechanisms by which specific immunobiotic strains enhance the recovery of granulopoiesis in immunocompromised hosts. Although the use of colony-stimulating factors can reduce the increased risk of infections induced by chemotherapy treatments, they are also the cause of several important side effects including bone pain, low-grade fever, and fatigue. Interestingly, the results expressed provide the basis for new applications of immunobiotics in order to stimulate the production of neutrophils and other types of leukocytes in the BM which would strength the ability of the host to fight against infections, without the side effects observed for stimulating factors. Certainly, this immunobiotic effect would improve the quality of life of patients receiving chemotherapy.

## Author Contributions

SS and SA wrote and approved the manuscript.

## Conflict of Interest Statement

The authors declare that the research was conducted in the absence of any commercial or financial relationships that could be construed as a potential conflict of interest.
